# An improved algorithm for flux variability analysis

**DOI:** 10.1186/s12859-022-05089-9

**Published:** 2022-12-19

**Authors:** Dustin Kenefake, Erick Armingol, Nathan E. Lewis, Efstratios N. Pistikopoulos

**Affiliations:** 1 Texas A &M Energy Institute, Texas A &M University, College Station, TX 77843 USA; 2 Department of Chemical Engineering, Texas A &M University, College Station, TX 77843 USA; 3 Department of Pediatrics, University of California, San Diego, La Jolla, CA 92093 USA; 4 Bioinformatics and Systems Biology Graduate Program, University of California, San Diego, La Jolla, CA 92093 USA; 5 Department of Bioengineering, University of California, San Diego, La Jolla, CA 92093 USA

**Keywords:** Flux variability analysis, Linear programming, Biological systems engineering

## Abstract

Flux balance analysis (FBA) is an optimization based approach to find the optimal steady state of a metabolic network, commonly of microorganisms such as yeast strains and *Escherichia coli*. However, the resulting solution from an FBA is typically not unique, as the optimization problem is, more often than not, degenerate. Flux variability analysis (FVA) is a method to determine the range of possible reaction fluxes that still satisfy, within some optimality factor, the original FBA problem. The resulting range of reaction fluxes can be utilized to determine metabolic reactions of high importance, amongst other analyses. In the literature, this has been done by solving $2n+1$ linear programs (LPs), with *n* being the number of reactions in the metabolic network. However, FVA can be solved with less than $2n+1$ LPs by utilizing the *basic feasible solution* property of bounded LPs to reduce the number of LPs that are needed to be solved. In this work, a new algorithm is proposed to solve FVA that requires less than $2n+1$ LPs. The proposed algorithm is benchmarked on a problem set of 112 metabolic network models ranging from single cell organisms (iMM904, ect) to a human metabolic system (Recon3D). Showing a reduction in the number of LPs required to solve the FVA problem and thus the time to solve an FVA problem.

## Introduction

Flux balance analysis (FBA) is an optimization based technique that predicts the steady-state fluxes of the reactions in a metabolic network at the optima of a biological imperative, such as production/consumption of biomass or a specific metabolite [1]. However the FBA problem does not always yield a unique solution for a given metabolic network. The range of these equally optimal (and perhaps sub-optimal) solutions is addressed with flux variability analysis (FVA) [2]. FVA is a technique to quantify the feasible ranges of reaction fluxes, *v*, of a metabolic network at optimal (or sub-optimal) production, given by FBA. To this end, FVA can be viewed as a generalization of FBA. The applications of FVA for metabolic networks, include medicine and health [3–5], understanding and improving the production of bio-fuels [6–9], and analyzing effects of mutation of bacterial strains [10, 11]. In essence, this technique allows for the analysis of the flexibility of reactions in a metabolic network. The FVA problem specifics are discussed in more detail in the "FVA overview" section.

Significant advances have been made for solving FVA problem on large metabolic networks, including the work of FastFVA and VFFVA for effective parallelization of the FVA problem [12, 13]. These methods are based on batching of the optimization problems to many CPU cores to maximize parallelization efficiency, and show significant performance increases over the nominal FVA algorithm. That means, they rely purely on parallelization to speed up solving $2n+1$ LPs problems. However, these algorithms do not make the problem less computationally expensive, the work is simply divided over more CPU cores. Notably, the computational burden can be improved by reducing the number of LPs that must be solved to obtain the FVA solution.

To this end, this work demonstrates a novel FVA algorithm to inspect the intermediate solutions of the optimization problems to explicitly reduce the number of optimizations that must be calculated for complete FVA of a metabolic network. This is done by motivating the solution inspection from the viewpoint of properties of optimal solutions of LPs. Then the algorithm is proposed that utilizes solution inspection to remove the necessity to calculate all $2n+1$ LPs. This algorithm is implemented and compared on real world metabolic networks against COBRApy, a state-of-the-art software package, for number of LPs evaluated and the time to solve the FVA problem [14].

### FVA overview

FVA is carried out in two phases, firstly a single LP is solved to find the maximum objective value, $Z_0$. This is the value that maximizes the biological imperative, $c^Tv$. Here, *c* is simply a vector of coefficients defining the biological value of each reaction, *v*. Phase 1 of FVA is identical to an FBA, but as this solution to the phase 1 problem (Eq. 1) is typically highly degenerate the range of fluxes, $v_i$ is determined in phase 2. FBA maps to the linear program (LP) found in Eq. 1. With $S \in \mathbb {R}^{\{m\times n\}}$, with *v* represents the fluxes of each reaction in the network, $\mu$ is the fractional optimality factor of the FBA objective $Z_0$, $c \in \mathbb {R}^n$ is the vector of coefficients denoting the biological imperative, with $\underline{v} \in \mathbb {R}^n$ and $\overline{v} \in \mathbb {R}^n$ are the bounds on lower and upper bounds on flux values respectively [12].

1a $$\begin{aligned}&Z_0 = \max _{v}\quad \quad c^Tv \end{aligned}$$

1b $$\begin{aligned}&\text {s.t.} \quad Sv = \mathbf {0} \end{aligned}$$

1c $$\begin{aligned}&\underline{v} \le v \le \overline{v} \end{aligned}$$

1d $$\begin{aligned}&v\in \mathbb {R}^n \end{aligned}$$

In phase two of the FVA calculation, the extents of fluxes of the metabolic network,$v_i$ are calculated. This is typically done by solving an additional 2*n* LPs with an additional constraint to either allow for suboptimality ($\mu < 1$) or enforce exact optimality of the FBA problem ($\mu = 1$) depicted in Eq. 2d. However, as will be discussed in the next section, not all of these sub-problems must be solved due to the proposed solution inspection procedure. This solution inspection procedure is the key step that reduces the need to calculate all 2*n* LPs that occur in phase two, and by doing so explicitly reduces the computational complexity of the entire problem.

2a $$\begin{aligned}&\max _{v}/\min _{v}\quad \quad v_i \end{aligned}$$

2b $$\begin{aligned}&\text {s.t.} \quad Sv = \mathbf {0} \end{aligned}$$

2c $$\begin{aligned}&c^Tv \ge \mu Z_0 \end{aligned}$$

2d $$\begin{aligned}&\underline{v} \le v \le \overline{v} \end{aligned}$$

2e $$\begin{aligned}&v\in \mathbb {R}^n \end{aligned}$$

## Algorithm overview

In this section a detailed description of the LP reduction procedure is presented, where the *basic feasible solution* property of LPs is used as a motivation to inspect intermediate LP solutions to reduce the number for LPs that must be solved during phase 2 of FVA. Then the proposed FVA algorithm is presented, where the typical FVA algorithm is augmented with solution inspection procedure. Some of the more specific implementation details of the proposed algorithm are outlined as well.

### Solution inspection

A well known property of bounded and feasible linear programs is that the optimal solution can be found at a vertex of the feasible space, this is known as the *basic feasible solution* (BFS) property [15]. The direct implication from this insight is that there must be an active set at the solution, $v^*$, with *at least* as many constraints as variables in the LP. The active set is simply the set of constraints where there is no slack between the solution, $v^*$, and the constraint boundary. In the FVA problem Eqs. 1 and 2, typically have a structure that implies that many bounds of flux variables (e.g. $\underline{v}$, and $\overline{v}$) must be active for any LP solution. If $Sv = \mathbf {0}$, has *m* equality constraints and there are *n* fluxes, and given $n > m$; any BFS of these LPs are constrained by some upper and lower bounds of *v*.

In essence, if we have a metabolic network with fewer metabolites than the number of reactions, then the FBA/FVA problem has less equality constraints than number of variables, and thus some of the flux values *must* be at either the upper or lower bound at a BFS. This insight leads to a simple procedure to check if solutions to any of the LPs, $v^*_i$, are at the upper and lower bounds at any LP. If a bound is attained for any flux variable at any LP solution in the FVA process then the LP associated with finding the bound of that flux, $v_i$ can be skipped, as it is already known that the bound is attainable. This is to say, if a flux variable is found at the maximum extent the variable can attain, there is no longer a reason to consider if there is a larger extend that could be obtained.

### Proposed FVA algorithm

In this section, the proposed algorithm for FVA is described. This is a prototypical implementation as described by [12], however this algorithm has been augmented with the solution inspection procedure applied to each intermediate LP solution. Thus allowing for a reduction in the number of optimization problems that need to be solved for FVA. The pseudo code of the proposed algorithm can be seen in Algorithm 1.
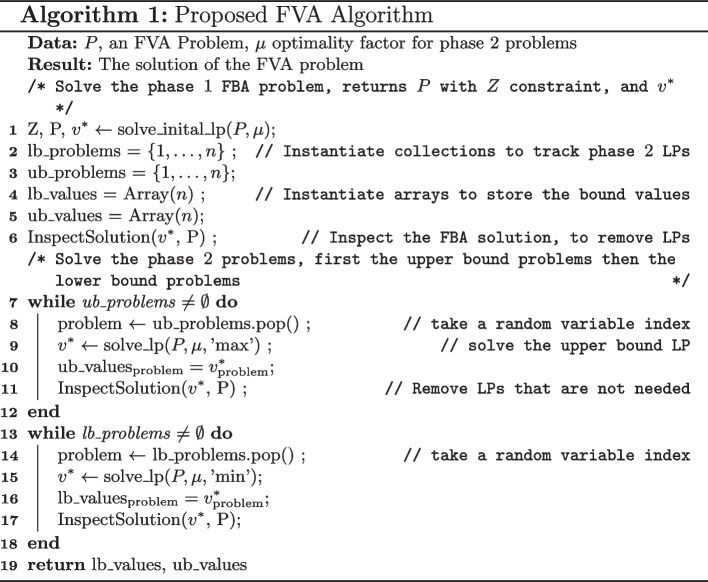


**Fig. 1:**
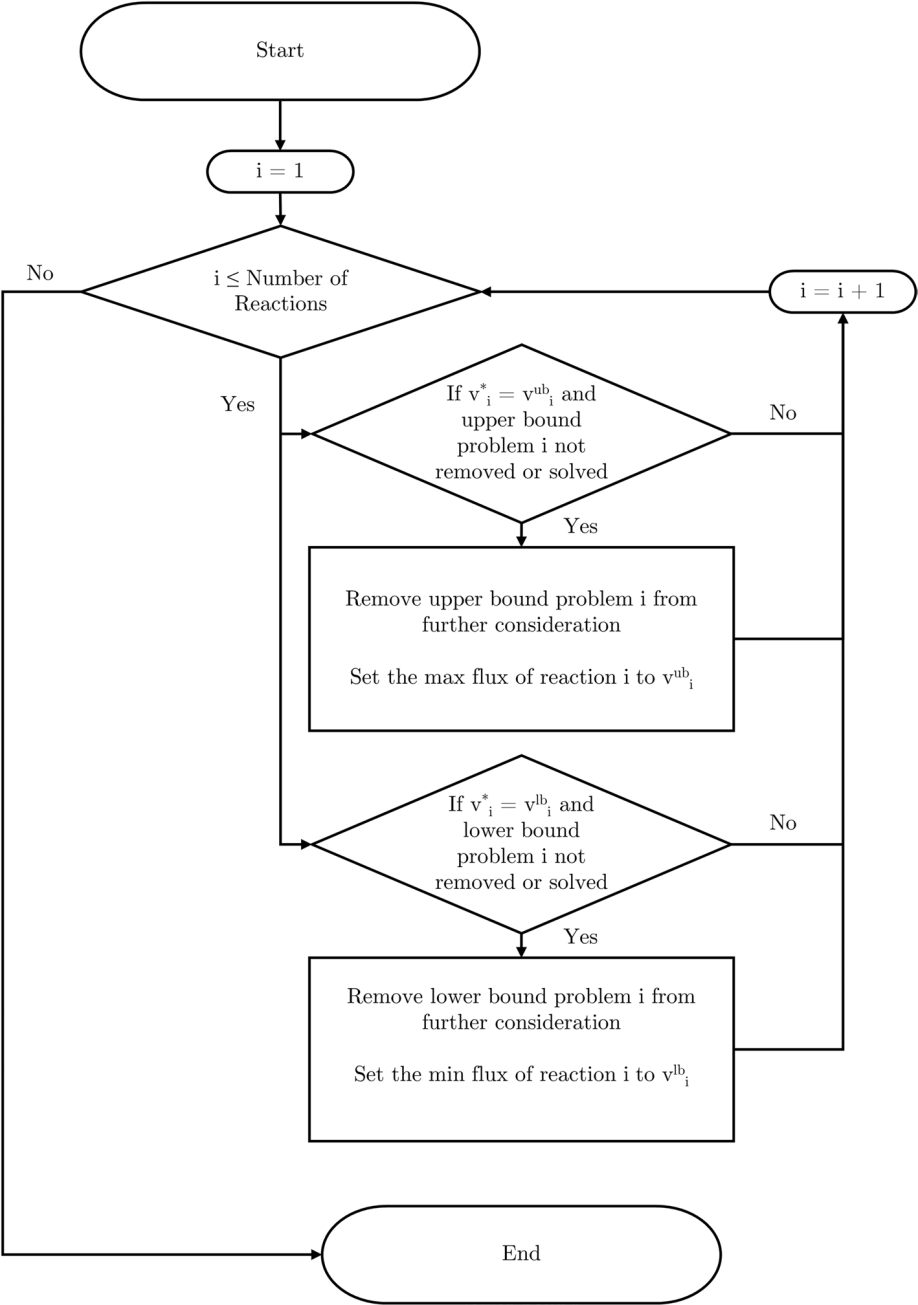
Solution inspection procedure for inspecting the solution of each LP, $v^*$ for the purposes of reducing the number of number of LPs that need to be solved for FVA

The solution inspection procedure can be simply described with the following; check if the solution to an LP, $v^*$, is at any of the upper and lower bound and remove the corresponding phase 2 problem from consideration if this problem has not been solved (or not removed yet). The pseudo code for this procedure can be seen in Algorithm 2 and a flowchart representation of this routine can be seen in Fig. 1. This solution procedure sales linearly with the number of reactions in the metabolic network, denoted as $\mathcal {O}(n)$, with *n* being the number of reactions. If it is taken into account that this procedure is called $2n + 1$ times, the overall time complexity of incorporating the solution inspection procedure into the FVA calculation is $\mathcal {O}(n^2)$, which has considerably lower time complexity than solving a single LP [15]. This analysis shows at least a theoretical basis of implementing the solution inspection procedure.
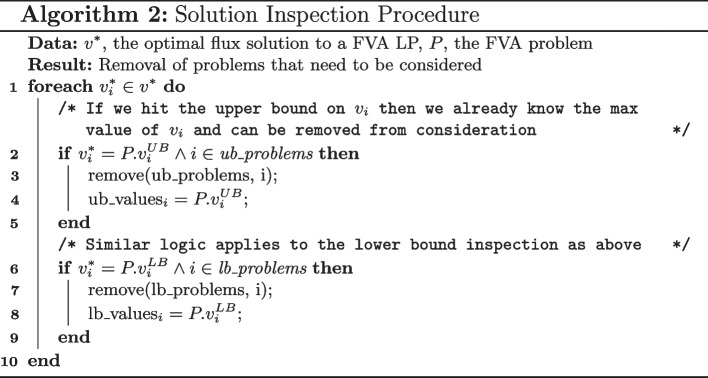


There are some specific implementation considerations to take into account when implementing FVA type algorithms. The LPs are recommended to be solved via the simplex method for two reasons. Firstly, in the case of degenerate LPs the simplex method grantees that the resulting optimal solution follows the basic feasible solution property. Additionally, the simplex algorithm the solution of the last LP can be used to warm start the next LP, as it avoids the initialization phase of the simplex algorithm, thus reducing the time to solve each individual LP. In particular, the primal simplex algorithm should be used over the dual simplex method. The reasoning behind this choice is that when changing the objective the last solution is not a feasible point of the dual LP [15] (except in the case where $c^T = e_i^T$, and only for one iteration of the second stage). A performance regression of 30–100% in time to solve was observed when using dual simplex when compared to primal simplex method as implemented in Gurobi 9.5.2 on the problems considered in the case study in the "Computational study" Section [16]. The reuse of the previous solution to warm start the next LP has been demonstrated in literature previously, notably in the method FFVA [12].

## Example problem

**Fig. 2:**
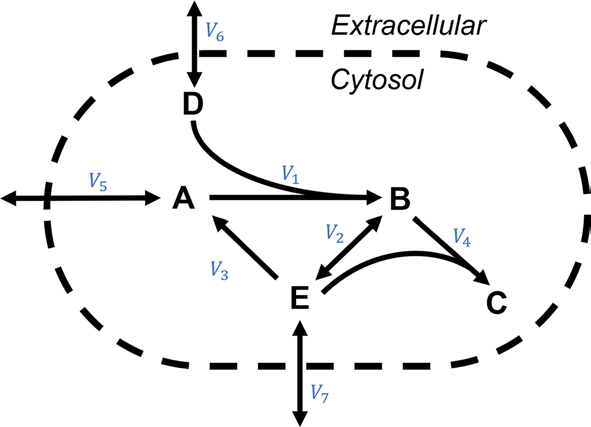
Graphical representation of the metabolic network of the example problem. In this system there are 7 reactions labeled as $V_1, \dots , V_7$ with 5 metabolites $\text {A}, \dots , \text {E}$. This network has two compartments, the extracellular region and the cytosol region. Figure adapted from [17]

In this section, the proposed FVA algorithm will be applied to a small metabolic network, adapted from [17]. This network model contains two compartments, with 7 reactions and 5 metabolites; this network proxies a simple bacterial metabolic network. A diagram of this system can be seen in Fig. 2. This problem will be used as an example of utilizing the solution inspection procedure to reduce the overall number of LPs required to solve the FVA problem.

In phase 1 of FVA, the FBA LP is solved as depicted in Eq. 3. For this problem, $Z = 0$ with $v^* = \mathbf {0}$. By inspection of the solution of the phase 1 problem, $v^*$, we can observe that the lower bounds of $v_1$, $v_3$, $v_4$, $v_5$, and $v_7$ were attained. As we already known that these variables can reach their lower bounds, we do not need to solve the LPs relating to finding their lower bounds. Similarly, as the FBA problem is no different from maximization of $v_4$ the LP to find the upper bound of $v_4$ is not needed as we can reuse this solution. In this example, by inspecting the phase 1 solution; we were able to remove 6 LPs from the total 14 LPs expected to be solved in phase 2 with the conventional algorithm.

3a $$\begin{aligned}&Z = \max _{v}\quad \quad v_4 \end{aligned}$$

3b $$\begin{aligned} \text {s.t. }\quad \begin{bmatrix} -1 &{}0 &{}1 &{}0 &{} 1 &{}0 &{}0\\ 1 &{}-2 &{}0 &{}-1 &{} 0 &{}0 &{}0\\ 0 &{}0 &{}0 &{}2 &{} 0 &{}0 &{}0\\ -1 &{}0 &{}0 &{}0 &{} 0 &{}1 &{}0\\ 0 &{}2 &{}-1 &{}-1 &{} 0 &{}0 &{}1\\ \end{bmatrix}v&= \textbf{0} \end{aligned}$$

3c $$\begin{aligned} \begin{bmatrix} 0&-10^5&0&0&0&-10^5&0 \end{bmatrix}\le v&\le \begin{bmatrix} 10^5&10^5&10^5&10^5&10^5&10^5&10^5 \end{bmatrix} \end{aligned}$$

With phase 1 complete, we now enter phase 2 of the FVA calculation. Here we calculate the range of $v_i$ within a $\mu$ factor of $Z_0$, e.g. $c^Tv \ge \mu Z_0$, here we consider $\mu =0.9$. This constraint is added to all LPs in phase 2. The structure of the phase 2 LP for this metabolic network can be seen in Eq. 4, with the added optimality factor constraint seen in Eq. 4c. In the case of this example problem this constraint is a entirely redundant, but is added for completeness. First, we solve for the remaining upper bound problems. We start with the upper bound of $v_1$ LP, with resulting solution $v^* = \langle 10^5, 5\times 10^4,10^5,0,0,10^5,0 \rangle$. We have $v_1^\text {max} = 10^5$, and in addition $v_3$, and $v_6$ attain their upper bounds, which removes the need to check their upper bounds via the LP procedure. This removes an additional 2 LPs from consideration. All other upper bound LPs result in the same solution, and thus do not remove additional LPs but confirm the upper bounds of the remaining flux variables. Out of the original 7 LPs needed to find upper bounds only 4 LPs were required after introduction of the solution inspection procedure.

4a $$\begin{aligned}&\max _{v}/\min _{v}\quad \quad v_i \end{aligned}$$

4b $$\begin{aligned} \text {s.t. }\quad \begin{bmatrix} -1 &{}0 &{}1 &{}0 &{} 1 &{}0 &{}0\\ 1 &{}-2 &{}0 &{}-1 &{} 0 &{}0 &{}0\\ 0 &{}0 &{}0 &{}2 &{} 0 &{}0 &{}0\\ -1 &{}0 &{}0 &{}0 &{} 0 &{}1 &{}0\\ 0 &{}2 &{}-1 &{}-1 &{} 0 &{}0 &{}1\\ \end{bmatrix}v&= \textbf{0} \end{aligned}$$

4c $$\begin{aligned}&v_4 \ge 0 \end{aligned}$$

4d $$\begin{aligned} \begin{bmatrix} 0&-10^5&0&0&0&-10^5&0 \end{bmatrix}\le v&\le \begin{bmatrix} 10^5&10^5&10^5&10^5&10^5&10^5&10^5 \end{bmatrix} \end{aligned}$$

The same procedure is carried out for the lower bound LPs. Only flux variables $v_2$ and $v_6$ are not known to exist on the lower bound, thus at most 2 LPs are needed to be calculated in this step instead of the original 7. Both LPs are solved but provide no further reduction in LPs. For the entire system FVA only 7 total LPs are needed with the proposed method, compared to the 15 required without inspecting LP solutions. This FVA analysis shows $v_\text {min} = \langle 0,0,0,0,0,0,0 \rangle$, and $v_\text {max} = \langle 10^5, 5\times 10^4,10^5,0,0,10^5,0 \rangle$ which is the same result as the standard FVA algorithm. This result shows at the optima, reactions 4, 5, and 7 have no net flux and are not necessary, at the optimal flux values identified by FBA.

## Computational study

In this section, we benchmark the proposed algorithm and a state-of-the-art implementation of the FFVA algorithm. For the purposes of this computational study; a problem set of 112 metabolic networks were created from the metabolic models available from the BIGG models repository and the non-redundant models considered in Guebila et al. [13, 18]. Each model representing a different problem in the problem set. These cover a broad spectrum of metabolic network size and types, including metabolic networks from *homo sapiens*, *Escherichia coli*, *Saccharomyces cerevisiae*, and *Trypanosoma cruzi*. A subset of the models utilized and their metabolic networks, are described in Table 1. These models were selected based selecting a diversity of species and network sizes.

The proposed FVA algorithm implementation is compared against COBRApy’s implementation of FVA [14]. Both the proposed algorithm and COBRApy are implemented in Python to present an equitable comparison for the time to solve an FVA problem. The benchmark is run on a Python 3.7 environment, using Gurobi 9.5.2 as the LP solver. These tests were carried out on a desktop with an i7-12700K CPU and 3200 MHz RAM in Windows 11. The process affinity for the benchmark was set to the P-cores of the CPU to minimize run to run variance in time to solve. In addition, as the proposed algorithm is implemented serially, COBRApy is set to run in serially as well.

**Fig. 3:**
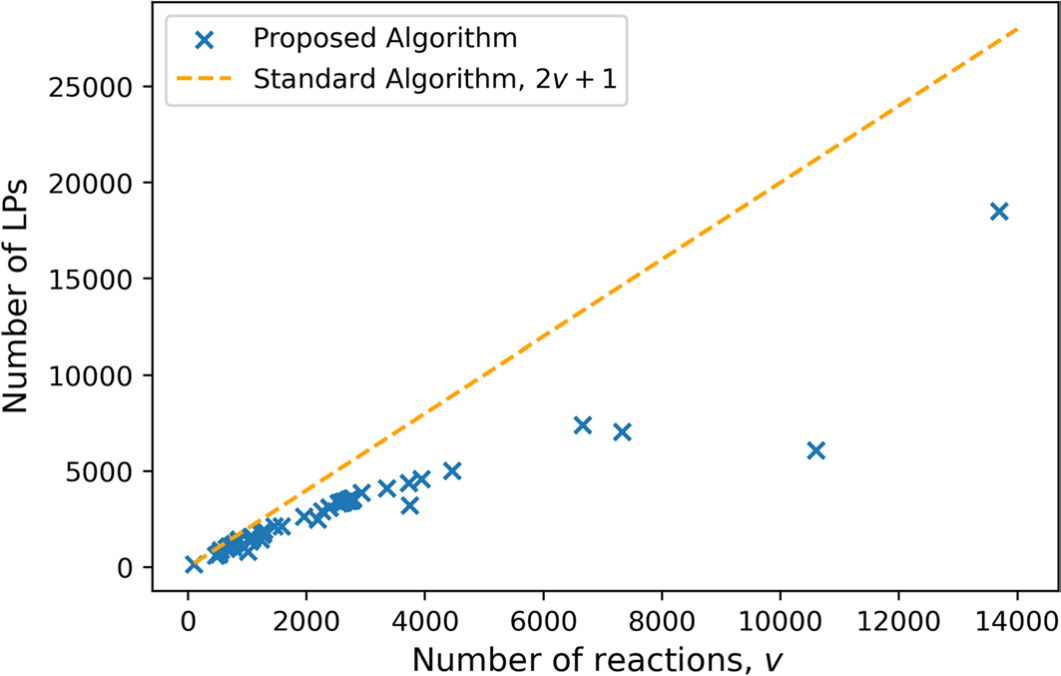
Comparison of the number of LPs required to solve w.r.t. the number of reactions in the model. I can be seen that there is a reduction in the number of LPs required to solve the FVA problem with the proposed algorithm compared to the nominal algorithm

**Fig. 4:**
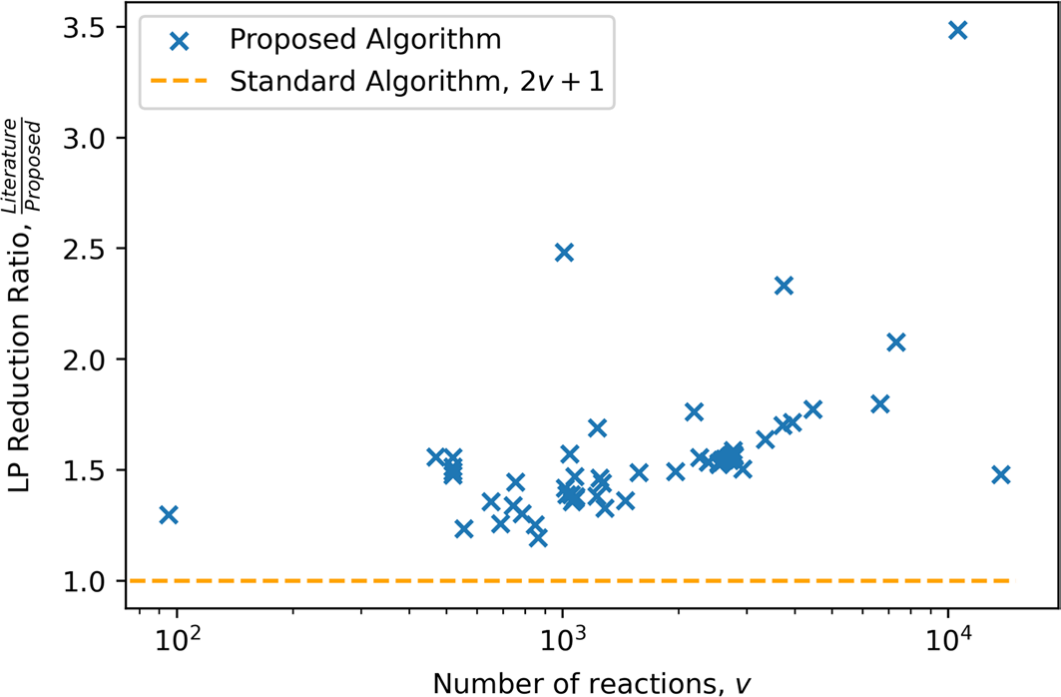
Comparison of the relative amount of LPs required to solve w.r.t. the number of reactions in the model. Marks above 1, indicate the reduction ratio of LPs. Here it can be seen that a general trend emerges with greater reduction ratios with larger metabolic networks, *v*

**Fig. 5:**
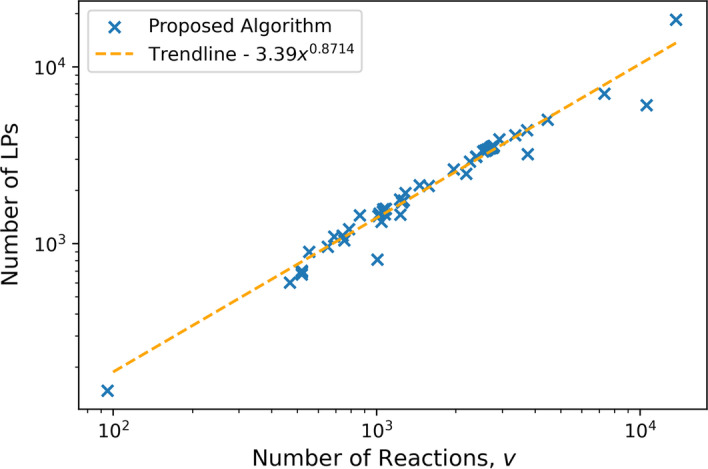
The trend of the number of LPs required to solve w.r.t. the number of reactions in the model. This shows an approximate reduction in the time complexity of solving FVA for the model systems considered, to approximately $\mathcal {O}(n^{\frac{7}{8}})$

**Fig. 6:**
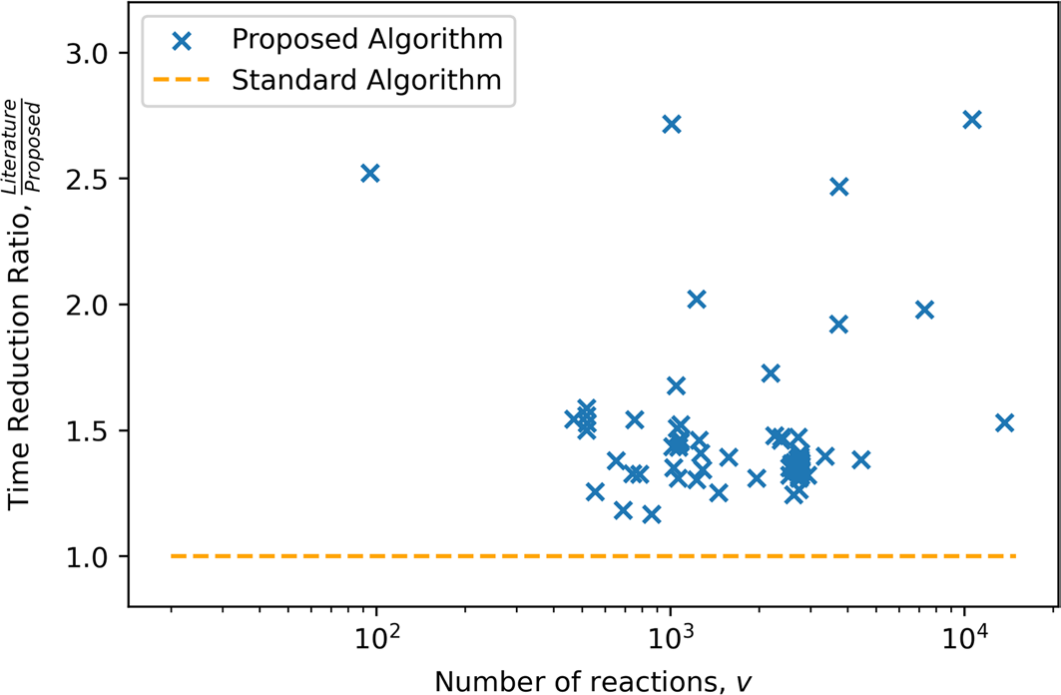
The ratio of time taken to solve the FVA problem, comparing the literature algorithm to the proposed algorithm. The trend is quite stochastic, this is due in part to the numerical complexity of some LPs being pruned in some cases while not being pruned in others

The primary comparison of interest is the number of LPs solved for any given FVA problem and secondly, the total solution time. As the purpose of the solution inspection step is to reduce the number of LPs solved, this is checked for each metabolic network in the problem set with promising results when compared to the nominal algorithm. It can be observed in Fig. 3, that every one of the considered 112 problems observed a reduction in the number of LPs required to solve. There is an apparent behavior that as the number of metabolites in a network increases the relative amount of LPs that are skipped are increased, as can be observed in Figs. 4 and 5. The specific number of LPs to solve the FVA problem for a selection of FVA problems can be observed in Table 2, where it can be seen that for every FVA problem considered that there was a reduction in the number of LPs. This demonstrates that the solution inspection procedure is capable of reducing the number of LPs that must be considered in FVA across a broad set of problem instances. Some models achieve, very strong results such as iAT_PLT_636 and Recon3D, both metabolic models for *Homo sapiens*. An LP reduction factor of $60\%$ and $71\%$ were achieved for these models respectively, with a time reduction of $63\%$ and $64\%$ respectively. As to be expected, by reducing the number of LPs that must be solved for the FVA problem the total time to solve was reduced for every problem in the problem set, as can be seen in Fig. 6. Selected problem time comparisons can be seen in Table 3. There is an apparent change in the time complexity of the FVA problem, of $\mathcal {O}(n)$, bringing the number of LPs that must be solved to $\mathcal {O}(n^{\frac{7}{8}})$, at least for this problem set. It can be seen as a general trend that as the problem size of the FVA problem grows that the effectiveness of the proposed algorithm increases when compared to the standard algorithm. This observation can be explained by the solution inspection procedure that is solved at each step of the algorithm, leading to larger problems having more opportunities for LPs being removed from consideration.

The premise that introducing the solution inspection procedure is confirmed, from the theoretical insight to the computational results. There is an overhead with interacting with the LP solver in the Python environment, for both submitting LPs to be solved and extracting the optimal solution vector, $v^*$, and could explain why the time reduction and the LP reduction factors are not in line when comparing against the implementation in COBRApy. In addition the LPs in FVA do not have the same complexity, and there is in general a large disparity in the computational complexity of each LP as has been noted by [13], which could contribute to this observation. The solution inspection procedure, given $v^*$, is quite fast and contributes to less then $5\%$ of the computational overhead of the proposed algorithm.

**Table 1:** Overview of the metabolic network models utilized in the computational study. Here we have a large range of model sizes with respect to the number of metabolites and reactions in the networks, and a diversity of originating species

Model name	Metabolites	Reactions
iAB_RBC_283	342	469
iAF1260b	1668	2388
iAT_PLT_636	738	1008
iEC1356_Bl21DE3	1918	2638
iEC1364_W	1927	2764
iECSP_1301	1920	2712
iIS312_Amastigote	606	519
iJN1463	2153	2927
iJO1366	1805	2583
iLB1027_lipid	2172	4456
iLJ478	570	652
iMM904	1226	1577
iNJ661	825	1025
Recon3D	5835	10600

**Table 2:** Comparison between the number of LPs solved with the proposed algorithm and the literature algorithm as implemented by COBRApy on some selected FVA problems

Model name	Proposed FVA algorithm LPs	COBRApy FVA LPs	Ratio
iAB_RBC_283	602	939	0.64
iAF1260b	3108	4777	0.65
iAT_PLT_636	812	2014	0.40
iEC1356_Bl21DE3	3499	5481	0.64
iEC1364_W	3525	5529	0.64
iECSP_1301	3489	5425	0.64
iIS312_Amastigote	686	1039	0.66
iJN1463	3890	5855	0.66
iJO1366	3361	5167	0.65
iLB1027_lipid	5024	8913	0.56
iLJ478	961	1305	0.73
iMM904	2120	3155	0.67
iNJ661	1477	2051	0.72
Recon3D	6082	21201	0.29

**Table 3:** Comparison between the proposed FVA algorithm and COBRApy FVA algorithm with time to solve on selected problems from the problem set. It should be noted that this time reduction is not uniform, some models such as iAT_PLT_636 and Recon3D show significantly higher reductions in solve time

Model name	Proposed FVA algorithm time (s)	COBRApy time (s)	Ratio
iAB_RBC_283	0.283	0.437	1.54
iAF1260b	7.230	10.653	1.47
iAT_PLT_636	1.114	3.02	2.71
iEC1356_Bl21DE3	8.791	12.03	1.37
iEC1364_W	9.443	13.02	1.38
iECSP_1301	8.676	11.85	1.37
iIS312_Amastigote	0.355	0.533	1.50
iJN1463	9.980	13.195	1.32
iJO1366	8.450	11.564	1.37
iLB1027_lipid	23.25	32.175	1.38
iLJ478	0.712	0.983	1.38
iMM904	3.465	4.830	1.40
iNJ661	1.538	2.081	1.35
Recon3D	74.86	204.82	2.73

## Conclusion

In this work, a FVA algorithm that does not rely on solving $2n+1$ LPs was presented. Instead of solving $2n+1$ LPs, a solution inspection procedure is used to remove the necessity to solve all $2n+1$ LPs. The proposed algorithm is then demonstrated on a small example problem that emulates a small metabolic network showing a reduction in the number of LPs from 15 to 7. This algorithm is then tested on a broad spectrum of real world metabolic networks and compared with a state-of-the-art implementation. The proposed algorithm demonstrated a reduction in the number of LPs required to solve the FVA problem and a reduction in the time to solve for these metabolic networks. For some organism wide metabolic models, speed ups on the order of $2\times$ and were observed, with an average speed up factor of 1.44 over the entire problem set. The algorithm implementation and the case study benchmark are open source under the MIT license and source code can be found at https://github.com/DKenefake/fasterfva.

In the future, incorporating the solution inspection with the dynamic parallelism could increase the computational performance and allow for increase in overall performance by utilizing more threads of execution. Additionally, alternative variations of FVA such as ’loop-less’ FVA could see similar performance uplifts via the intermediate solution inspection approach [19, 20]. In the loop-less FVA case, the sub problems are mixed integer linear programs (MILPs), removing the necessity of solving every sub problem would significantly decrease the computational time, as MILPs are more computationally challenging. Incorporation of this algorithm into the COBRApy Toolbox is a goal of the authors, so that this algorithm can be utilized by other researchers. This methodology can be extended to the general case of redundant constraint removal and tightening, where the BFS property of LPs can be extended to reduce the number of optimization problems required to generate a minimal non-redundant constraint set. These will be addressed in forthcoming works.

## Data Availability

All models sourced for the case study are available at http://bigg.ucsd.edu/models and https://bmcbioinformatics.biomedcentral.com/articles/10.1186/s12859-020-03711-2.

## Abbreviations

LP: Linear programming
MILP: Mixed integer programming
FBA: Flux balance analysis
FVA: Flux variability analysis
FFVA: Fast flux variability analysis
VFFVA: Very fast flux variability analysis

## References

[1] Watson MR (1984). Metabolic maps for the Apple II. Biochem Soc Trans.

[2] Burgard AP, Vaidyaraman S, Maranas CD (2001). Minimal reaction sets for escherichia coli metabolism under different growth requirements and uptake environments. Biotechnol Prog.

[3] Pentjuss A, Rubenis O, Bauze D, Aprupe L, Lace B (2013). Flux variability analysis approach of autism related metabolism in stoichiometric model of mitochondria. Biosyst Inf Technol.

[4] Asgari Y, Khosravi P, Zabihinpour Z, Habibi M (2018). Exploring candidate biomarkers for lung and prostate cancers using gene expression and flux variability analysis. Integr Biol.

[5] Asgari Y, Khosravi P (2020). Flux variability analysis reveals a tragedy of commons in cancer cells. SN Appl Sci.

[6] Hay J, Schwender J (2011). Metabolic network reconstruction and flux variability analysis of storage synthesis in developing oilseed rape (*Brassica napus* l.) embryos. Plant J.

[7] Pentjuss A, Kalnenieks U (2014). Assessment of zymomonas mobilis biotechnological potential in ethanol production by flux variability analysis. Biosyst Inf Technol.

[8] Hay J, Schwender J (2011). Computational analysis of storage synthesis in developing *Brassica napus* l. (oilseed rape) embryos: flux variability analysis in relation to 13c metabolic flux analysis. Plant J.

[9] Wang FS, Wu WH (2020). Computer-aided design for genetic modulation to improve biofuel production.

[10] Bushell ME, Sequeira SI, Khannapho C, Zhao H, Chater KF, Butler MJ, Kierzek AM, Avignone-Rossa CA (2006). The use of genome scale metabolic flux variability analysis for process feed formulation based on an investigation of the effects of the zwf mutation on antibiotic production in streptomyces coelicolor. Enzyme Microb Technol.

[11] Khodayari A, Maranas CD (2016). A genome-scale escherichia coli kinetic metabolic model k-ecoli457 satisfying flux data for multiple mutant strains. Nat Commun.

[12] Gudmundsson S, Thiele I (2010). Computationally efficient flux variability analysis. BMC Bioinf.

[13] Guebila MB (2020). Vffva: dynamic load balancing enables large-scale flux variability analysis. BMC Bioinf.

[14] Ebrahim A, Lerman JA, Palsson BO, Hyduke DR (2013). Cobrapy: consraints-based reconstruction and analysis for python. BMC Syst Biol.

[15] Luenberger DG, Ye Y (1984). Linear and nonlinear programming.

[16] Gurobi Optimization, LLC: Gurobi optimizer reference manual 2022. https://www.gurobi.com.

[17] Cuevas DA, Edirisinghe J, Henry CS, Overbeek R, O’Connell TG, Edwards RA (2016). From dna to fba: How to build your own genome-scale metabolic model. Front Microbiol.

[18] King ZA, Lu J, Dräger A, Miller P, Federowicz S, Lerman JA, Ebrahim A, Palsson BO, Lewis NE (2015). BiGG models: a platform for integrating, standardizing and sharing genome-scale models. Nucleic Acids Res.

[19] Noor E, Lewis NE, Milo R (2012). A proof for loop-law constraints in stoichiometric metabolic networks. BMC Syst Biol.

[20] Schellenberger J, Lewis NE, Palsson BØ (2011). Elimination of thermodynamically infeasible loops in steady-state metabolic models. Biophys J.

